# A new intranasal therapy (MP29-02*) is more effective than current firstline therapy regardless of season, symptom or severity.

**DOI:** 10.1186/2045-7022-5-S4-P38

**Published:** 2015-06-26

**Authors:** Claus Bachert, Wytske Fokkens, Peter Hellings, Glenis Scadding, Ullrich Munzel, Joaquim Mullol

**Affiliations:** 1 Ghent University Hospital, Dept of Oto-Rhinolaryngology, Ghent, Belgium; 2 Academic Medical Center, Department of Otorhinolaryngology, Amsterdam, Netherlands; 3 University Hospitals Leuven, Dept of Otorhinolaryngology, Head & Neck Surgery, Leuven, Belgium; 4 The Royal National Throat Nose and Ear Hospital, London, UK; 5 Meda, Corporate Clinical Affairs, Bad Homburg, Germany; 6 Hospital Clinic IDIBAPS, CIBERES, Barcelona, Spain

## Background

Moderate-to-severe allergic rhinitis (AR) is often poorly controlled. Patients remain symptomatic on treatment, despite multiple therapies. A more effective treatment is needed. We assessed the efficacy of MP29-02* (a novel intranasal formulation of azelastine hydrochloride (AZE) and fluticasone propionate (FP) in an advanced delivery system) during different seasons and for different symptoms and severities vs AZE, FP or placebo (PLA).

## Method

Four thousand and twenty two moderate/severe SAR patients (≥12 yrs) were randomized into 4 double-blind, PLA-controlled, 14-day, parallel-group trials to MP29-02*, AZE, FP or PLA nasal sprays (1 spray/nostril bid), during the Texas mountain cedar (MP4001), Spring (MP4002), Autumn (MP4004) and Spring/Summer (MP4006) seasons. Overall change from baseline (CFB) in reflective total nasal symptom score (rTNSS) was the primary endpoint. It was assessed by severity post-hoc in 2 ways (more severe AR: median baseline rTNSS >18.9 or median baseline RQLQ >3.9; less severe AR: median baseline rTNSS ≤18.9 or median baseline RQLQ ≤ 3.9). CFB in individual nasal and ocular symptom scores was also assessed.

## Results

The response to MP29-02* was consistent across seasons; mean CFB -5.5, -5.5, -5.6 and -5.6 in each study (p<0.001 vs PLA). Nasal symptom relief was significantly greater with MP29-02* than with FP or AZE in all studies. In study MP4001 MP29-02 was approx. twice as effective as FP (relative difference (RD) 47%; p=0.0031) and 3 times as effective as AZE (RD: 66%; p<0.0001). For less severe AR (defined by median baseline rTNSS) the RD to MP29-02* was 42% vs FP (p=0.0188) and 64% vs AZE (p=0.0002), increasing to 49% and 70% vs FP (p=0.0436) and AZE (p=0.0035), respectively for more severe AR. When severity was categorized according to median baseline RQLQ the RD to MP29-02* was 60% vs FP (p=0.0244) and 69% vs AZE (p=0.0068) for those with less severe AR compared to 49% vs FP (p=0.0194) and 64% vs AZE (p=0.0013) for those with more severe AR. MP29-02* provided superior relief from all nasal and ocular symptoms than AZE or FP, which was particularly evident for congestion (p=0.0034 vs FP; p=0.0001 vs AZE) & ocular itching (p=0.0240 vs FP; p=0.0033 vs AZE).

## Conclusion

MP29-02* provides consistently superior symptomatic relief to an intranasal antihistamine or topical corticosteroid in AR patients regardless of season, symptom or severity, supporting MP29-02 as the drug of choice for AR.

*Dymista

